# Long-Term Condition Self-Management Support in Online Communities: A Meta-Synthesis of Qualitative Papers

**DOI:** 10.2196/jmir.5260

**Published:** 2016-03-10

**Authors:** Chris Allen, Ivaylo Vassilev, Anne Kennedy, Anne Rogers

**Affiliations:** ^1^ NIHR CLAHRC Wessex Faculty of Health Sciences University of Southampton Southampton United Kingdom

**Keywords:** social media, patient online communities, long-term conditions, chronic disease, self-management, self-care, illness work, social networks, qualitative meta-synthesis

## Abstract

**Background:**

Recent years have seen an exponential increase in people with long-term conditions using the Internet for information and support. Prior research has examined support for long-term condition self-management through the provision of illness, everyday, and emotional work in the context of traditional offline communities. However, less is known about how communities hosted in digital spaces contribute through the creation of social ties and the mobilization of an online illness “workforce.”

**Objective:**

The aim was to understand the negotiation of long-term condition illness work in patient online communities and how such work may assist the self-management of long-term conditions in daily life.

**Methods:**

A systematic search of qualitative papers was undertaken using various online databases for articles published since 2004. A total of 21 papers met the inclusion criteria of using qualitative methods and examined the use of peer-led online communities for those with a long-term condition. A qualitative meta-synthesis was undertaken and the review followed a line of argument synthesis.

**Results:**

The main themes identified in relation to the negotiation of self-management support were (1) redressing offline experiential information and knowledge deficits, (2) the influence of modeling and learning behaviors from others on self-management, (3) engagement that validates illness and negates offline frustrations, (4) tie formation and community building, (5) narrative expression and cathartic release, and (6) dissociative anonymity and invisibility. These translated into a line of argument synthesis in which four network mechanisms for self-management support in patient online communities were identified. These were (1) collective knowledge and identification through lived experience; (2) support, information, and engagement through readily accessible gifting relationships; (3) sociability that extends beyond illness; and (4) online disinhibition as a facilitator in the negotiation of self-management support.

**Conclusions:**

Social ties forged in online spaces provide the basis for performing relevant self-management work that can improve an individual’s illness experience, tackling aspects of self-management that are particularly difficult to meet offline. Membership in online groups can provide those living with a long-term condition with ready access to a self-management support illness workforce and illness and emotional support. The substitutability of offline illness work may be particularly important to those whose access to support offline is either limited or absent. Furthermore, such resources require little negotiation online because information and support is seemingly gifted to the community by its members.

## Introduction

Population aging has resulted in an increased prevalence of long-term conditions, which has resulted in increased expenditure on the provision of care for those affected [1,2]. As a consequence, self-management has become an increasingly important paradigm in health care delivery and the promotion of self-management of long-term conditions is now an enduring feature of health care policy [3-6]. This meta-synthesis of qualitative papers seeks to explore the self-management of long-term conditions in the relatively new context of online communities.

The current economic and philosophical landscape of the National Health Service (NHS) necessitates the need for illness work to be delegated to those with a long-term condition and policy makers hope this will reduce health service utilization [7] and improve health outcomes [2,3,5]. The need for self-management is reinforced by the fact that those living with a long-term condition spend very little time engaged with health care professionals compared to the time spent on activities that are required to manage their condition in daily life [1,8].

Recent research has begun examining the social context of long-term condition self-management and, more specifically, the role of others in shaping and supporting self-management practices [1,7,9,10]. In particular, research conducted by Vassilev et al [9] demonstrate the importance of work in long-term condition self-management, particularly in respect of one’s illness work force, those in one’s network who provide assistance in the self-management of their condition through illness, everyday, and emotional work, which can include a biographical dimension [9,11,12]. Types of illness work suggested by Vassilev et al [9] and Rogers et al [1] include:

- Illness (specific) work: work such as taking medication, taking and interpreting measurements, understanding condition and its symptoms, and making appointments [9]
- Everyday work: tasks such as housekeeping, occupational labor, support, and activities relating to diet and exercise, shopping, and personal care [9]
- Emotional work: work related to comforting when worried/anxious about everyday matters, such as health, well-being, and companionship (including a biographical dimension relating to the reassessment of personal expectations, capabilities, future plans, personal identity, relationships, and biographical events) [9]
- Contingency/improvisation: the work involved in getting things back on track [1]
- Translation/mediation: the work involved in translating abstract knowledge into practical knowledge that can then be implemented [1]
- Coordination: the negotiations and renegotiations in the ways in which work is done, such as what work is done, by whom, when, how, and why [1]
- Advocacy work: work done by others on one’s behalf [1]

Weak social ties also contribute to illness work by affording greater access and transmission of information between network members; the value of these ties lays in their quantity rather than their intensity [8].

Online communities are particularly good at facilitating the creation of weak ties [13-16]. As a result, community membership may afford people a larger, more diverse social network than would otherwise be available because ties mediated online are not restricted by temporal, spatial, or geographical limitations that typically define offline social networks [16,17]. Those with more diverse social networks are said to self-manage their long-term condition better compared to those with fewer social ties [10]; however, this has only been explored in the context of offline social networks and there is a clear need to better understand the role of online contacts in the self-management of long-term conditions.

An existing review by Ziebland and Wyke [18] conceptualizes seven domains through which patient experiences online influence health. These domains were finding information, feeling supported, maintaining relationships, affecting behavior, experiencing health services, learning to tell the story, and visualizing the disease [18]. Although this review was useful in framing the landscape of peer-to-peer support online due to its focus on understanding the exchange of experiential information on health, it did not specifically focus on long-term condition self-management. Moreover, the changing landscape of online communities in relation to the proliferation in the ways in which people access them makes them more relevant to our daily lives because ties mediated online are now more immediately available [19,20].

In this context, its relevant to understand the extent to which social ties created in these online spaces contribute to long-term condition self-management through the negotiation of illness work (illness work is described as the visible and invisible activities of long-term condition self-management) [9]. In recent years, the study of self-management support has introduced a focus on the mechanisms of networks that mediate self-management support for long-term conditions and the influence this has on the mobilization of resources [1,7,9,21]. Although there has been increasing awareness of the significance of the Internet as a forum for support and engagement for self-management support [18], previous studies have not specifically focused on the mechanisms of such networks and how they may mediate long-term condition self-management support. Offline, three mechanisms linking social networks and health-related outcomes exist: sharing knowledge and experience within a community, access and mediation of resources, and an awareness and ability to deal with network relationships [21]. It is clear that in offline networks, those with a long-term condition need to be able to navigate their personal social networks and negotiate and renegotiate existing relationships [21]. Although Vassilev et al’s [21] article successfully demonstrates the negotiation of self-management support in traditional offline social networks, these specific aspects have not been explored in terms of online communities. Thus, this meta-synthesis aims to generate an elaborated understanding of the negotiation of self-management support and illness work in patient online communities for those with a long-term condition. This is relevant for informing the design of online interventions.

## Methods

### Qualitative Meta-Synthesis

A meta-synthesis draws on the subjective and interpretive nature of existing qualitative research to construct more complete and plausible understandings of reality than what is currently available from the existing literature. There are several approaches to qualitative synthesis; in this instance, Paterson et al’s [22] process of meta-synthesis was used. Like secondary analysis, qualitative synthesis involves reinterpretations, but the analysis is generated from already existing published findings of other authors [23]. Such data exist in the form of first- and second-order constructs [22]. The first-order constructs represent direct feedback from the study participants and the second-order constructs represent the key findings of the researchers [22]. The third-order constructs relate to the interpretation of the findings of the articles based on the synthesized first- and second-order constructs [22]. Paterson et al [22] explains this process by stating that: “The authors of primary research reports have constructed the research findings in accordance with their own understanding and interpretation of the data” [22] (p.6); subsequently, “The meta-synthesists have constructed an aggregated account based on their own interpretations of the primary researchers’ constructions. Consequently, the meta-synthesists deals with constructions of constructions” [22] (p. 7). As such, the process moves beyond the findings of the original papers to generate more complete understandings of the phenomena being investigated because it pulls together and makes use of concepts derived from multiple studies, using a wide variety of methods, contexts, and interpretive frameworks [21,22].

By including articles that used different methods, examined different types of online communities, and different conditions, this meta-synthesis is able to add to the existing evidence base, bringing research data from an initially narrow focus (ie, a specific condition and online community) toward a broader interpretation of long-term condition illness work in online settings.

### Inclusion/Exclusion Criteria

To guide the systematic search of the literature, the research team (CA, IV, AK, AR) agreed on the following predetermined inclusion and exclusion criteria, taking into account the aims of the meta-synthesis. The predetermined inclusion criteria were (1) studies examining the use of online communities for those with a long-term condition (including communities hosted on social media sites such as Facebook and Twitter), (2) studies that focused on online communities from a naturalistic open setting, (3) research between 2004 (the year the term “Web 2.0” became popularized) and 2015 (when the search took place), and (4) research that used qualitative methods. The predetermined exclusion criteria were (1) studies not written in English, (2) research including interventions, (3) research from the perspective of health care professionals/carers/relatives, (4) research that only used quantitative methods, (5) literature reviews and review papers, letters to the editor and editorials, commentaries and feature articles, dissertation theses, reports, conference papers, and abstracts, (6) studies only on traditional Internet use and without an interactive social component (ie, Web 1.0 and blogs), and (7) studies with a commercial, advertising, or marketing focus, where levels of bias could be seen as high.

### Search Strategy

A systematic approach was used to locate the relevant published research studies in the area of online communities and long-term conditions. Because online communities in relation to health have been explored across a multitude of professional and theoretical concepts, health, social care, psychology, and sociology databases were searched. The systematic search of the research literature used the following databases: Allied and Complementary Medicine Database (AMED), Cumulative Index to Nursing and Allied Health Literature (CINAHL), the Cochrane Database of Systematic Reviews, DelphiS, EMBASE, the International Bibliography of Social Sciences (IBSS), MEDLINE, PsycINFO, Scopus, Sociological Abstracts, and Web of Science. The searches were conducted using a predetermined search strategy, using the search terms in (Textbox 1).

**Figure 1:**
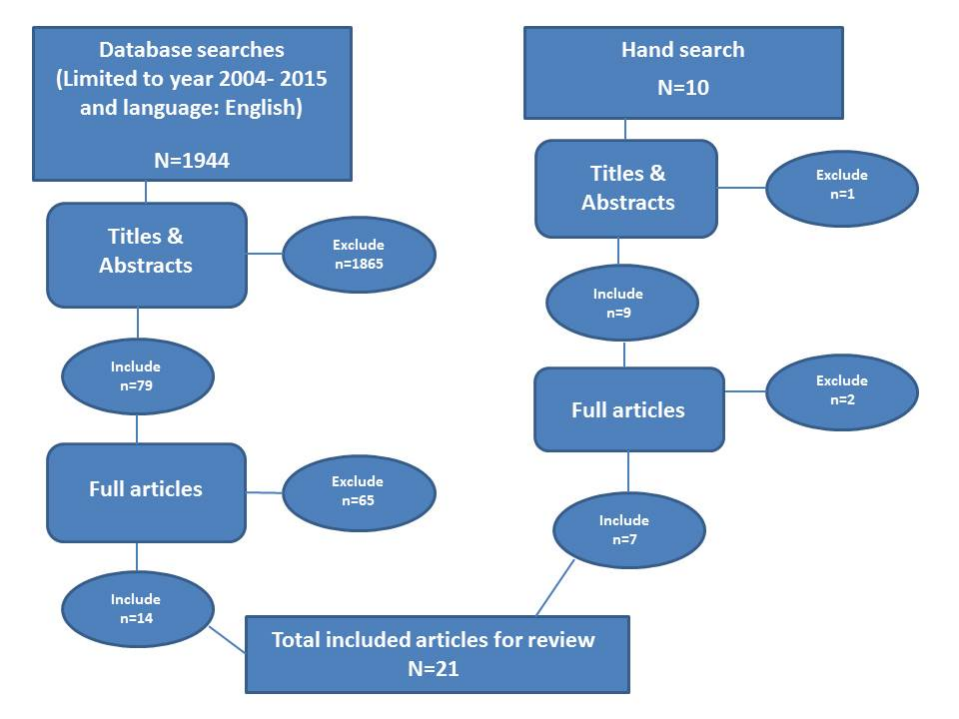
Flowchart of systematic search strategy, process and selection of research papers for review.

The systematic review of the available literature occurred in August 2015. The search strategy using the aforementioned databases located 1944 research articles. Titles and abstracts were reviewed against the inclusion criteria; from this, hard copies of 79 articles were obtained. These were screened against the inclusion/exclusion criteria (by CA, AK and IV), resulting in a total of 14 papers. A further 10 papers were found through submersion in the research literature and through the reference lists of eHealth articles read by the research team. From this, a further seven papers met the criteria for inclusion. All selected papers were discussed by the team in view of the objectives of better understanding the contribution of online social networks in long-term condition self-management. This process can be seen in Figure 1 and a summary of the included articles can be seen in Table 1.

**Table 1:** Articles included in the meta-synthesis and quality appraisal scores using the Critical Appraisal Skills Programme (CASP) tool.

Study	Condition	Platform	Method	Sample	Study details	CASP score^a^
Attard and Coulson [24]	Parkinson disease	Disease-specific discussion board/forum	Qualitative thematic analysis of messages posted to a discussion board	1013 messages posted to the board between 2003-2010	To explore the experiences of members of a Parkinson’s disease forum	9
Barker [25]	Fibromyalgia	Disease-specific discussion board/forum	Thematic analysis	249 participants in Fibrospot	Examines the conflicts between lay and expert knowledge in electronic support groups	9
Brown and Altice [26]	Opioid dependence	Disease-specific discussion board/forum	Grounded theory approach	121 threads from 13 discussion boards in a 26-month period	To identify facilitators of self-treatment by online buprenorphine/naloxone users	9
Coulson [17]	Alcohol use disorder	Disease-specific discussion board/forum	Inductive thematic analysis-netnography	738 messages on 3 UK-based discussion boards	To explore in-depth how members of online alcohol use disorder communities engage with peer-to-peer support	9
Coursaris and Liu [27]	HIV/AIDS	Disease-specific discussion board/forum	Content and thematic analysis	5000 postings(not disclosed how many participants contributed to this)	To provide an in-depth understanding of social support exchanges in online HIV/AIDS self-help groups	8
Greene et al [28]	Diabetes	Facebook	Content analysis	233 wall posts and 457 discussion topics	Examine the content of communication in Facebook communities dedicated to diabetes	8
Hadert and Rodham [29]	Arthritis	Disease-specific discussion board/forum	Interpretive phenomenological approach	60 users who posted 87 initial messages + 314 users who posted 981 replies	To discover how and why the online arthritis message board was used	9
Kazmer et al [30]	ALS	Patients Like Me (an online community that connects people with the same condition)	Inductive thematic analysis	1000 randomly selected messages from an available 2500 messages posted between Feb 2006-Nov 2008	How and why knowledge is shared among the distributed participants in the PLM-ALS threaded discussion forum	9
Kirk and Milnes [31]	Cystic fibrosis	Disease-specific discussion board/forum	Online ethnographical approach	279 individuals who participated in forum over a 4-month period	To explore how online peer support is used by young people and parents to support self-care in relation to cystic fibrosis	9
Loanne and D’Alessandro [32]	Motor neuron disease/ALS	Disease-specific discussion board/forum	Content analysis	499 posts made by 133 participants	Explores whether social capital can exist in an online health community for people affected by MND/ALS	8
Matura et al [33]	Pulmonary hypertension	Disease-specific discussion board/forum	Qualitative descriptive methodology	Convenience sample (all posts in 2010)	To determine how patients with pulmonary hypertension use online discussion boards	9
Mazzoni and Cicognani [34]	Systematic lupus erythematosus	Disease-specific discussion board/forum	Content analysis	118 posts corresponding to 118 authors	To explain the demand/supply of social support through the Internet in relation to the description of personal illness experience	9
Merolli et al [35]	Chronic pain	Did not specify; patients recruited through Facebook, Twitter, Daily Strength, and Patients Like Me	Thematic content analysis; online survey	218 people with chronic pain who completed an online survey	To examine what social media therapeutically affords people with chronic pain who are self-managing their condition	9
Mo and Coulson [36]	HIV/AIDS	Disease-specific discussion board/forum	Thematic analysis of completed online surveys	115 participants who completed an online survey	To explore the potential empowering and disempowering outcomes of online support group use by those with HIV/AIDS	9
Rodham et al [37]	Complex regional pain syndrome	Disease-specific discussion board/forum.	Interpretive phenomenological analysis	60 participants who posted or commented on a post on a discussion forum in a 4-month period	To explore how an online message board designed for patients and carers of patients with CRPS was used; specifically, sought to explore the exchanges that took place on the online message board	10
Van Berkel et al [38]	ALS, diabetes, ADHD	Disease-specific discussion board/forum	Deductive thematic analysis	5532 posts from seven message boards	To examine whether empowerment processes occur on message boards discussing medicines used to treat three chronic conditions as well as examining the quality of information that is shared	9
Van Uden-Kraan et al [39]	Fibromyalgia, arthritis, breast cancer	Disease-specific discussion board/forum	Content analysis of postings to a discussion board/forum	Random sample of 1500 postings to discussion board/forum for fibromyalgia, arthritis, breast cancer	To explore who uses online support groups, what topics are discussed, and what self-help mechanisms are used in these groups	8
Van Uden-Kraan et al [40]	Fibromyalgia, breast cancer, arthritis	Disease-specific discussion board/forum	Semi-structured interviews, inductive analysis	32 participants	To explore if, and in which ways, patients feel empowered by participation in patient online communities	9
Wentzer and Bygholm [41]	COPD and fertility problems	Disease-specific discussion board/forum	Qualitative analysis using critical interpretation and narrative analysis	4301 posts to 2 forums	Is communication in online patient support groups a source of individual and/or collective empowerment?	8
Willis [42]	Arthritis	Disease-specific discussion board/forum	Ethnomethodology	20 members across 4 communities	To understand how patient with arthritis use patient online communities to exchange illness related information to better manage their long-term condition	9
Zhang et al [43]	Diabetes	Facebook	Case study	Case study of a Facebook group with 30,000 users	Explores Facebook as a platform for health information and communication, specifically what the characteristics of the Facebook diabetes group and its members	8

^a^ Maximum score is 10.

**Predetermined search terms.**

“Social media” OR “Social network* site*” OR “web 2.0″ OR “Health 2.0″ OR “discussion board*” OR “discussion forum*” OR “forum*” OR “online support group*” OR “electronic support group*” OR “online communit*” OR “patient online communit*” OR “facebook” OR “twitter” OR “tweet*” OR “myspace” OR “patientslikeme” OR “patients like me” OR “second life”

AND

Chronic” OR “Chronic disease*” OR “Chronic Illness*” OR “Long term condition*” OR “Long-term condition*” OR “Long term health condition*” OR “LTC*” OR “chronic pain*” OR “pain*” OR “fibromyalgia” OR “chronic obstructive pulmonary disease” OR “COPD” OR “diabet*” OR “irritable bowel syndrome” OR “IBS” OR “heart disease” OR “HIV” OR “AIDS” OR “Stroke”

AND

Self-management” OR “self management” OR “Self-care” OR “Self care”

### Quality Appraisal

The included papers were critically appraised according to the Critical Appraisal Skills Programme (CASP) checklist for qualitative research (by CA) [44]. The checklist allows qualitative research evidence to be appraised systematically, guiding the reviewer about the results, their validity, and their transferability [44]. The results can be seen in Table 1 and demonstrate the included articles represented high-quality research; therefore, they were all included in the analysis.

The findings of this synthesis are limited by the methodology of many of the included papers [17,24-34,37-39,41-43], which used either “netnography” (a specific form of ethnography adapted to computer-mediated communities) [45] or other approaches that did not directly engage participants nor did they provide consent toward participation in the study. Although the approach of using the comments of others from public online communities without their specific consent is considered ethical by current British Psychological Society (a representative body for psychology and psychologists in the United Kingdom) guidelines [46], it meant that it was not possible to observe more intimate encounters (eg, direct messaging, email, texting, telephone conversations, or even meeting offline) that may have emerged over time. This meant the behavior of participants was not affected by the presence of a researcher in the community, but it also meant that only what members elected to post could be used as research data.

Only three articles [35,36,40] specifically engaged network members. It is possible that because these papers directly engaged those using these communities that they permitted a greater discussion of how people experienced them. Thus, they were perhaps more likely to discuss the negative and the positive aspects of community membership. It may have been that in the other articles, those with bad experiences were less likely to post negative experiences, such as flaming (a hostile online interaction) caused by toxic disinhibition, which led to people being rude or angry toward others in ways that they would not be offline [47,48]. This kind of behavior had the potential to make people feel personally attacked if they expressed opinions that were different to other members [36]. Additionally, these articles were perhaps more likely to demonstrate concerns about misinformation (eg, people sharing inaccurate or harmful information) and people presenting themselves as experts [35,40]. Therefore, to some extent the positive feel of the other articles may be a result of their methodology; however, there is no shortage of articles that have identified the potential harms [49-57] and ethical issues [57,58] surrounding online communities.

## Results

The long-term conditions examined in relation to online communities were diverse and clearly projected different illness experiences. They included heavily stigmatized conditions such as alcohol and substance use disorders [17,26] and human immunodeficiency virus and acquired immune deficiency syndrome (HIV/AIDS) [27,36]; medically contested conditions, such as fibromyalgia [25,35,39,40]; and extremely physically disabling conditions, such as Parkinson disease [24], arthritis [29,39,40], chronic obstructive pulmonary disease (COPD) [41], cystic fibrosis [31], and motor neuron disease [30,32].

### Patient Online Communities’ Involvement in Long-Term Condition Self-Management: Second-Order Synthesis of Concepts

To synthesize the data, the articles identified were read and logged into extraction forms (by CA). The extraction form used was adapted from a previous meta-synthesis. These were used to ensure the multiple concepts in the included articles were translated into one another. The extraction form included demographics, condition, group type, principal research question/aims, methodology/data collection strategy, principal findings, subthemes, theoretical concepts, conclusions, and study limitations. Within these extraction forms, we also included all verbatim quotes from participants (first-order constructs); this allowed us to see that the quotes from the participants fitted logically into the second-order constructs (the original author’s interpretations) of the original articles.

Because the second-order constructs are interpretive, the concepts across the articles are presented in different ways. To synthesize the findings and concepts of the different articles into one another (second-order synthesis), we experimented with different visualizations of the second-order constructs used in the existing articles and examined the different arrangements of the key concepts from these studies. This involved a number of iterations before the final conceptualization of second-order constructs were agreed (by CA, IV, AK, AR). Following the synthesis of the second-order constructs, six second-order constructs were identified that illuminated how the social connections forged online contributed to long-term conditions self-management. From this, the synthesized second-order constructs (taken from translating the key themes in the included articles) were brought together and then reconfigured as a line of argument toward better understanding the negotiation of illness work in patient online communities.

#### Redressing Offline Experiential Information and Knowledge Deficits

Members were frequently drawn to online groups through an unmet offline need for condition-specific information that was easy to understand [40], could be customized to their specific needs [26,28,40,42], was based on patient experience [30,36,43], and was freely available at their convenience [36]. The need for accessible, accurate, and up-to-date information was often directed by inadequate access to information offline, whereby community members felt let down by information providers in their offline worlds [29,36]. This was often fuelled by time restraints and power relationships experienced in offline consultations, which appeared to inhibit information seeking [29]. Membership to an online community appeared to be a useful way of mitigating this, by affording members with greater access to information [28,29,36,39]. Network members were able to use these online communities to filter and navigate condition-specific information created by peers, in accessible language, at their convenience. This allowed the redressing of information asymmetry by affording individuals information their health care professional (HCP) did not feel they needed, withheld from them, or provided in a format they did not understand [36].

The information available in the groups frequently pertained to lived illness experience [25,28,30]. Members favored this information over the presumed expert knowledge of HCPs, whereby validity was bestowed on embodied illness experience [25,28,30]. Indeed, posts would insinuate that “expert patients” had a higher degree of condition-specific knowledge than HCPs [30]. These expert patients were able, through community action and shared knowledge, to assist others to locate information elsewhere [27,30] (both online and offline). Although some had concerns about the validity of the information posted [40], the information was frequently validated using a process of community vetting [28,43] with members intervening when bad information was posted [40]. This suggests that membership in these communities facilitates improved health literacy and resource navigation by pooling the collective knowledge and lay expertise of its members who have a vested interest in better understanding their condition [27,28].

#### The Influence of Modeling and Learning Behaviors From Others on Self-Management

The included articles all demonstrated online communities’ ability to enable members to reach out to peers for practical illness-specific advice. The peers that they connected with were able to develop expertise about daily treatment practices through trial and error, giving them valuable knowledge and information about the daily practicalities of self-managing a long-term condition that extended beyond the empirical evidence available to HCPs [28,30,42]. This afforded members an enhanced understanding of how to integrate multifaceted treatment regimens to balance the complexities of self-management in daily life [28,30,31,42]. Users learned from the self-management approaches of others by observing their self-management strategies, discovering new and more efficient strategies, and subsequently testing out these new strategies with their peers [42]. From this, they were able to select an approach that best met their needs [42].

The sharing of experiential information in online communities is an important feature in shaping the experience of those living with a long-term condition because the information shared in these communities frequently favored patient-centered goals as opposed to HCP-centered metrics [28,29,31]. This information was easier for members to configure to their specific needs and was less rigid than the information and self-management strategies provided offline [26,28,29,31,42].

#### Engagement Which Validates Illness and Negates Offline Frustrations

Having access to the online community made members feel less alone and provided a reference for what was a normal illness experience [17,24,25,29,31,36,40]. Members, who often lacked solidarity offline, were able to build a collection of symptoms into a shared identity [24,25,31,33,34]. Offline, members found it difficult to get a real understanding from friends and family and were able to use these online spaces to express these frustrations with a network of people who seemingly understood the challenging nature of self-managing their illness [25,29,34,36,37,40]. This was particularly the case in communities for conditions that lacked visible external cues or for which the somatic nature of the illness was contested [25,29,35]. This disparagement strengthened group solidarity and allowed users to feel validated and believed through engaging and identifying with other network members [25,29].

Meeting people who understood the challenging nature of self-management allowed members the opportunity to be positively appraised for accomplishments that their offline contacts might not recognize as achievements [28,37]. Members were commended for the achievement of smaller self-directed goals as opposed to ones set by HCPs [28,37]. This worked to motivate group members to believe in treatment recommendations, shared beliefs, and practices, thus encouraging treatment compliance [24,41].

The sharing of condition narratives enabled members the opportunity to reevaluate their situation through lateral and downward social comparison. Being able to see how others coped with their condition reassured members that they could manage their condition through education, adjustment, adaptation, and acceptance [17,29,33,35,40,42].

#### Tie Formation and Community Building

Communities often demonstrated a clear sense of comradery, with the communities inferring strong community structures, cultural norms, and group orthodoxies [24,28,31,32,40,43]. Many of these communities appeared to promote a positive, inclusive culture, bringing people of diverse backgrounds together to meet a shared purpose [24,32,36,43]. This sense of belonging, coupled with a shared lived experience of the condition and frustrations with offline support, facilitated the creation of friendships [24]. This creation of community led to members integrating the community into their everyday lives [32,40]. Members used endearing terms such as “family” and “friends” and would frequently engage in non-condition-related conversations, suggesting that the communities had facilitated strong bonds between members [24,27,36,40] with relationships evolving into offline spaces [36,40], where tangible benefits, such as offers of accommodation, could be realized [36].

In several instances, users connected with these communities to mitigate loneliness and isolation in their offline worlds [32,35,40], which appeared to be particularly important in instances where the disabling nature of the condition had led to an erosion of offline support and a reduced ability to form social ties in offline settings [32,35,40]. Often, network members faced clear social disadvantage in their offline worlds, but online belonged to lively, vivacious communities with resources of information and support offered freely as a public good to community members [32].

#### Narrative Expression and Cathartic Release

These communities provided a safe environment for the sharing of condition narratives. The process of narrative sharing offered immediate psychological relief because members often felt unable to express negative emotions offline due to the perceived need to maintain a positive social front [29,37]. Some members found sharing experiences easier online, preferring to talk to strangers online about their illness experience than with their offline contacts [35,36,40]. These online spaces provided them with a community of people ready to listen to their concerns and provide them emotional support and refuge [35,36,40]. Because these communities made members feel more able to openly express their need for support, they were possibly more likely to receive it and it is perhaps unsurprising that some users felt more supported online [29].

#### Dissociative Anonymity and Invisibility

Acquiring certain types of sensitive information, that may be important in developing a holistic self-management strategy such as information pertaining to sex and incontinence, appears to be easier to navigate in these online communities due to the presence of benign disinhibition and dissociative anonymity [47]. This appears to have an empowering effect by allowing members to ask questions that they would otherwise be too embarrassed to seek in their physical worlds [24,36].

### Understanding the Significance of Negotiating Self-Management Support and Illness Work in Online Communities: Third-Order Synthesis

**Figure 2:**
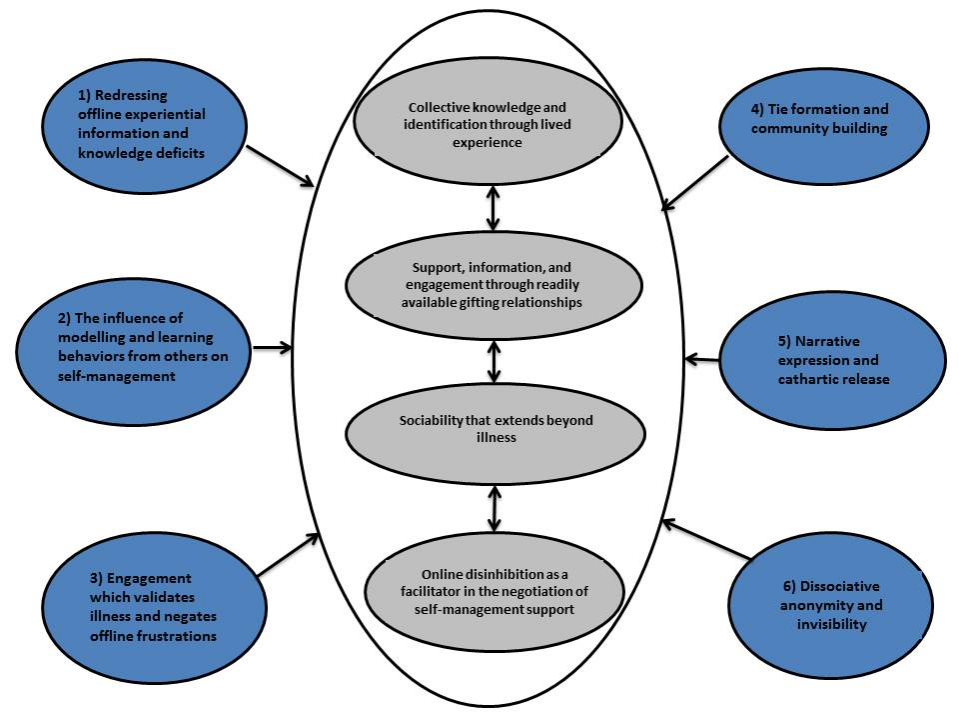
Summary of second- (blue) and third-order (gray) constructs in relation to the negotiation of self-management support in patient online communities.

Following a process of synthesis, the second-order constructs described previously were reconfigured toward understanding what is significant about the negotiation of self-management support and illness work in online communities for those living with a long-term condition. This translated into a line of argument synthesis in which four network mechanisms for self-management support in online communities were identified. A summary of the second- and third-order constructs is shown in Figure 2. In exploring the significance of online support networks compared to traditional offline ones, we drew on previous research examining the social context of long-term condition self-management and the network mechanisms involved in negotiating illness work [1,7,9,21]. This allowed us to examine whether similar mechanisms of self-management support exist and are mobilized online.

#### Collective Knowledge and Identification Through Lived Experience

Given that “the Internet has changed people’s relationship with information” [59] (p. 1), it is perhaps unsurprising that the ability of these communities to provide information featured highly in the included articles. Information and actionable advice based on lived experience contained highly specialized forms of experiential information that was unobtainable offline. These communities facilitated patient empowerment by affording members the right to explore the self-management of their condition in the context of their daily lives. This patient empowerment perspective, facilitated by membership in these groups, promoted a fundamentally different set of roles for HCPs and patients, whereby the collective knowledge created through lived experience was seen as more useful in the self-management of a long-term condition in daily life than medical knowledge. In line with previous research, these communities appear to foster empowerment and the potential to change the relationship between HCPs and patients, from one of compliance to one of shared ownership [60,61].

In addition to the availability of cocreated experiential knowledge, the presence of distributed health literacy enabled community members to find the information they required. Online social ties can act as health literacy mediators [62] in a process of distributed health literacy between network members, allowing people to benefit from the health literacy of others in their network, who may give them greater access to the information needed to manage their condition.

In offline consultations, a mediator such as close friend or family member is often present to help the person comprehend what is being discussed [7] and individuals can capitalize on the resources and links made with members of their social networks offline [62]. People’s knowledge about their condition is often shaped by others with the same condition in their personal networks [7,21]. However, this resource may not be available to everyone, such as in rare conditions or in situations where open discussion is difficult. People appear to be able to substitute offline information deficits with online contacts, whereby community members benefit from the health literacy skills of their peers. Examples included network members assisting in resource navigation [27] and explaining medical terminology [29,36,43].

Additionally distributed knowledge and information in these communities constituted a by-product of the continued engagement of network members [33,34]. Communities generated value through members “cocreating their own service encounter” [34] (p.167). Members were able to select the features that they required and request, receive, or search for information at their convenience [34]. Unlike offline encounters, a permanent record is made, which allows members to benefit from cumulative experiential information generated over time [30,32]. For some, the sheer volume of information available made it difficult to find the specific information they needed [40], which further signposts the need for community members to assist in the navigation of resources in these communities. Essentially, the strength of these communities drives the availability of the information. Many communities have a defined core group of members [63,64], whose informational and experiential knowledge can be disseminated to other members who may be less well informed. As Lester et al [65] hypothesized, although not everyone in the group knows as much as this expert core, they do know how to access expert members, who in turn know how to access information.

The provision of information should be considered an important component of any long-term condition self-management package [66], but information on its own has been found to have very little or no effect on self-management [67] and it is these communities’ ability to tie information to real-life parables that is most fascinating. Each individual’s needs are highly specific; therefore, self-management support must be tailorable. Members of these communities felt restricted by a lack of flexibility, choice, and control in self-management strategies dictated by HCPs, but could use these communities to observe the practice of others, adapting their self-management strategy to meet a specific problem or a change in their condition [28,35] through navigating the available information and deciding the approach which best met their needs [42]. This is perhaps why the coconstructed authoritative knowledge of community members built around the lived experience of self-managing a long-term condition in daily life was so valuable. Patient online communities appear to deliver a highly individual experience through the cocreation of community content. For example, it is perhaps unlikely that the traditional patient education perspective model of information would be able to disseminate highly experiential information, such as how someone with diabetes can count carbohydrates to enable drinking sessions without risking ketoacidosis [28], but such facets of information are clearly useful to someone with diabetes wanting to self-management their condition.

In addition to information and health literacy mediation, these communities facilitate the negotiation of illness emotional work and its biographical dimension, whereby emotional work relates to the provision of comfort when someone is upset, anxious, or worried about everyday issues, such as their health, well-being, and companionship [1]. Biographical dimensions of emotional work are associated with the revision of expectations, capabilities, plans, identity, relationships, and biographical events [1], and these online communities have a role in the realization of these components, particularly in allowing members the opportunity to reframe their lives [17,29,33,35,40,42].

By engaging in online communities, individuals were able to gain emotional support that they had been unable to access in their offline worlds and by connecting with those with a shared embodied experience were able to feel normal [17,24,25,29,35,36,40]. Through collective identification, these groups facilitated engagement, allowed individuals to make sense of their situation, and allowed them to receive positive appraisal for successful self-management practices [28,37]. Furthermore, through lateral and downward social comparison, these online communities allowed members the opportunity to compare their illness narratives with one another, enabling them to reassess their expectations, capabilities, and plans, while empowering them to realize that successful self-management is achievable [17,29,33,35,40-42]. Thus, through collective identification and engagement, these online communities provided the opportunity for validation, reassessment, and appraisal. But, for some, this was upsetting because it made many negative aspects of the disease visible, some of which they may not have considered [40].

#### Support, Information, and Engagement Through Readily Available Gifting Relationships

In addition to navigating network contacts, those with a long-term condition need to negotiate and renegotiate existing relationships, roles, and engagement with network members. Negotiating help offline is frequently accompanied by obligations and expectations and may be restricted by time [21]. Such obligations and expectations were not visible in the online communities explored here. Requests for help (resource mobilization) were rarely targeted at a specific network member; often requests for assistance were to the group as a whole, leading to many replying. This information is frequently gifted, with no reciprocal expectation, making help less tangible but potentially easier to obtain online than off.

In much the same way as gifting relationships stock UK blood banks [68], members of these networks gift these communities with information and support freely [29,32,35,36,39,43]. Much like donating blood, the decision to volunteer information cannot “of course, be characterized by complete, disinterested, spontaneous altruism” [68] (p. 89). Information and emotional labor is gifted to these communities by its members, who are potentially motivated to do this through a sense of obligation or through some awareness of need. Like donating blood, there may be “some expectation and assurance that a return gift may be needed and received at some future time” [68] (p. 89). In this sense, these online communities operate much like a gift economy with information and support being freely given, with little expectation of reciprocation, but fuelled by the desire that someone else may find the information useful and the pride of building a community [28,32,34-36].

The process of sharing information appeared to have a useful dual purpose, providing information for those in need, but also affording others with their altruistic need to impart the knowledge that they had accumulated [28,32,34-36]. Being able to offer information that others may find helpful appeared to foster feelings of validation and self-worth, feelings that are often suppressed by illness [35]. The voluntary provision of information was part of these groups’ culture and occurred more frequently than in response to direct questioning. Although offline peer mentors have benefited from providing support through finding meaning and social reinforcement of their own self-management behaviors, gift exchange in these online communities is different to that in offline support groups. Offline, the process of sharing has been found to improve the internal capacity of individuals to cope with stress and can be a motivating factor in long-term condition self-management through mediating lifestyle changes and affording new self-management tools [21]. However, these gift exchanges may fail offline because the recipients of the intended gift may not turn up. Because of the asynchronous nature of the Internet, members can post information and support that others may benefit from at a later time. Furthermore, these gifts have the potential to benefit anyone who accesses the group, whereas this kind of gift offered offline can only benefit those physically present because no lasting record is made of the encounter. Because of the giving nature of such communities, there is a wealth of information and support that requires little or no negotiation.

#### Sociability That Extends Beyond Illness

In these communities, conversations frequently extended beyond illness into everyday matters and interests [29,36,40], which seemed to provide “social hooks” for continued community involvement. Although people appear to migrate into these online communities due to offline information and emotional deficits, it is perhaps these hooks that result in continued engagement. Members spent time relaxing in these online communities [40] and enjoyed being able to socialize [35], which appeared to be particularly appreciated in circumstances where the presence of illness had led to the erosion of offline contacts [35,40]. Members looked forward to their online interactions with one another and enjoyed telling others about their day: “I have just got in from a lovely evening and couldn’t wait to get on and see if there was any mail for me...I thought I would share with you the events of the evening” [29] (p.189). For many, engagement with these online communities had become part of their daily routine: “You should really see it as a book. You’re in the middle of a story. And when you put the book down at night, you really want to continue reading the next morning.” [40] (p.409). These communities accompanied members throughout their day [40] and this may become increasingly important in the future as smartphones continue to integrate these technologies into our daily lives [69].

The presence of a long-term condition may place greater salience on support from family and close friends, reducing the opportunities to build and maintain contacts that extend beyond this. Socializing with people online and being able to build relationships with new people allows individuals to build new networks of influence that extend beyond intimate offline contacts. Consequently, those whose condition may have eroded the ease with which they can build and maintain weak social ties appear to benefit from being able to substitute for this by building new networks of contacts in patient online communities. However, that this support often remained online was a source of frustration for some who wanted to extend their relationship into offline spaces, but were restricted by geography [24]. Despite this, the ability to proactively extend networks that may have been eroded by the presence of a long-term condition is important because research suggests that those with a larger network of contacts consisting of both friends and family typically have the most favorable outcomes [70].

The “Internet paradox” article contains an argument that the Internet, as a social technology, may reduce socialization and psychological well-being [71]. Such concerns were voiced in Mo and Coulson’s [36] article: “...I noticed that my real-life relationships were declining due to the time I invested in the online community” [36] (p. 990). However, being able to access these communities enabled those whose social ties had been eroded through illness [35,37] to build new opportunities for sociability: “Through fibromyalgia you lose a lot of personal contact. Because you can’t go to birthday celebrations anymore, because you forget things, you’re often too tired and so on. And in this way you can rebuild your social contacts” [40] (p. 412). These communities may allow members to reach out to peers when offline socialization is not possible. Thus, being able to access peers online has the potential to mediate feelings of isolation and loneliness. Later research by Kraut et al [72] into the Internet paradox found that although those using the Internet generally experienced positive effects on social involvement, communication, and emotional well-being, the extent to which these benefits were realized was associated with offline support, whereby extroverts with good preexisting offline social networks fared better than introverts with reduced offline support. Additionally, research by Kuss and Griffiths [73] found that extroverts use social media for social enhancement, whereas introverts use them as a means of social compensation [73]. Although these findings were not in the context of patient online communities, it does suggest that introverts managing a long-term condition in these online communities may be distanced from offline social networks able to provide more tangible support in spite of being able to use the Internet to access a more diverse network.

#### Online Disinhibition as a Facilitator in the Negotiation of Self-Management Support

Being able to act anonymously online highlights the presence of managing moral identity work operating in these communities. Those with a long-term condition may decide that the need to be both independent and autonomous is so important that they choose not to activate offline support despite it being available [21]. As such, these online communities may protect offline relationships and allow those living with a long-term condition to negotiate illness work while remaining both independent and autonomous.

Suler’s [47,48] theory of an online disinhibition effect suggests that people behave differently on the Internet due to the presence of:

1. Dissociative anonymity: people may feel that their online actions cannot be attributed to their person. In a process of dissociation, people may feel they do not own their online behaviors.
2. Invisibility: online, people know that others do not know what they look like. This may make people feel more able to do things on the Internet that they would not do offline.
3. Asynchronicity: online interactions often do not occur in real time. Not having to cope with someone’s immediate reaction to something that has been said or done may disinhibit people.
4. Solipsistic introjection: the absence of face-to-face cues may alter normal self-boundaries. Because people cannot see what others look or sound like online, they may introject others into their own psyche.
5. Dissociative imagination: people may feel the online world is not real and that the people they interact with online are not real people.
6. Minimization of status and authority: there is often an absence of authority figures online and people may feel they can act more freely.

Dissociative anonymity, invisibility, and the minimization of status and authority appear to have a positive impact on the negotiation of self-management support online in the included articles. The presence of “benign” disinhibition appears to facilitate the negotiation of self-management support in patient online communities because people may be reluctant to seek certain types of support in their offline worlds due to societal and self-stigmatizations. Although the online disinhibition effect may explain some of the harmful behaviors driven by toxic disinhibition, which was visible in some of these communities [36], the disinhibiting nature of online communication appears to be mostly positive in allowing people to reach out to others for self-management support.

People are able to move around the Internet anonymously [47,48]. In some of these groups, people reveal their identity, but many used pseudonyms. As Suler [47,48] highlights, the Internet gives people the opportunity to separate their offline persona from their online actions. As such, through a process of dissociation, “the online self becomes a compartmentalized self” [47] (p. 322) and, in the context of patient online communities, appears to allow people to reach out to peers for information and emotional support without endangering their offline self. Suler [47,48] suggests that this can facilitate rapid or falsely intimate relationships, which might explain why such strong bonds appear to form in these online communities. Talking about stigmatized conditions is challenging offline. These online communities enable people to talk about their illness while remaining anonymous: “...at the time I wasn’t capable nor [ready] to approach an [AIDS service organization] nor disclose my status. I had so many guilty questions that I needed to talk to someone who would not know anything about my life nor recognize me” [36] (p. 987).

Even when everyone’s identity is known, people can feel invisible online [47,48]. This is protective and facilitates the negotiation of self-management support. Because online communication lacks nonverbal cues, people don’t have to worry about how they look or sound [47]. They can write, examine, and edit posts before sending, allowing complete control over disclosures and expressions. This editorial control is lacking in offline communications. This disclosure scrutiny and editorial freedom can lead to people feeling more comfortable discussing even everyday matters online [74]. Community members felt empowered to disclose due to this increased control: “this is an excellent medium for me to be able to control my interactions” [35]. But because of the lasting record associated with computer-mediated communication, some were skeptical in spite of this increased control: “I do not want to disclose my personal and painful journey via a social network site for it to be highlighted by others and ‘used’ as a way to finish me in my job” [35].

Additionally, online communication lacks nonverbal cues so people do not have to worry about nonverbal responses, such as frowns, shaking of heads, or other nonverbal signs of disapproval [47,48] that may inhibit offline disclosures. Offline, when people discuss emotional matters, they often avoid eye contact. These online communities offer “a built-in opportunity to keep one’s eyes averted” [47] (p. 322), thus avoiding awkward moments in which “the rheumatologist sneers a bit” [40] (p. 410).

The presence of benign disinhibition generates group resources because it facilitates conversations about stigmatized or taboo subjects that others may find useful and validating. It also provides a safe and effective environment for the negotiation of support, allowing people to freely discuss personal and/or embarrassing health narratives, which may be particularly important to those whose condition is heavily stigmatized as well as potentially enabling those with less stigmatized conditions to ask questions about more sensitive aspects of living with a long-term condition [17,47]. For some, these online communities represent the only place where information and support for self-management can be negotiated: “Only they know that I have HIV and my doctor, nobody else. They are my virtual family” [36] (p. 988).

## Discussion

This research strengthens our socialized understanding of long-term condition self-management by taking into account the illness work of social ties mediated online and the role such ties may have in the management of a long-term condition in daily life. Effective self-management support utilizes resources and networks that are available in the everyday lives of those with a long-term condition, which operate outside of formal health care, and this meta-synthesis has shown that these are available online and important to people. Those with a long-term condition appeared to reach out to these online communities because of an unmet offline need for information and/or emotional support. This substitutability of illness work has been seen before in offline social networks [7]; in this instance, it clearly signposts the importance of these online communities in negotiating illness work particularly when access to support offline is absent or limited.

It is clear that these communities afforded many benefits that have the potential to positively shape someone’s experience of living with a long-term condition. To some extent, the findings of this meta-synthesis necessarily overlap with the work of Ziebland and Wyke [18]. Certainly, the facility of these online communities to help people find information, feel supported, maintain relationships, experience health services, learn to relate, visualize their disease, and affect behavior [18] were all visible in the included articles and may all help to positively shape self-management. The distinction between this paper and that of Ziebland and Wyke [18] is the specific focus on the contribution of online social networks to long-term condition self-management illness work and the affordances of community membership rather than the impact of online patients’ accounts of experiences with health and health care.

This meta-synthesis has demonstrated that there are several benefits to members of patient online communities over and above those available to people simply searching for the experiential accounts of others. Membership of these online communities affords those living with a long-term condition ready access to a self-management support illness workforce, particularly in relation to illness and emotional work. However, in contrast to offline social ties, these online communities provide social ties that require significantly less maintenance, less reciprocation, and are easier to negotiate. This is potentially due to the presence of benign disinhibition and the gifting economic relationships of these online spaces, whereby information and support is donated freely, as a public good, with no immediate expectation of reciprocation. Unsurprisingly, everyday work appears largely absent in online self-management support perhaps due to the need for physical presence to assist in household tasks, shopping, or personal care. There is some suggestion in the research literature of relationships evolving into more intimate communication channels and offline spaces [36,40]; therefore, it is not unreasonable to suggest that “everyday work” may emerge in these relationships over time.

Importantly, social ties forged in online spaces can perform self-management work that can improve an individual’s illness experience and can reach areas that are particularly difficult to navigate offline. Because of this, patient online communities appear to be a promising place for the negotiation of self-management support for long-term conditions that may supplement and support offline information and support and should be included in future studies exploring the social context of long-term condition self-management.

This study had a few limitations from which future directions for research are suggested. The majority of the included articles examined patient online communities that existed on condition-specific discussion forums and boards. In contrast, newer apps, such as Facebook and Twitter, are poorly represented in the existing research literature with no existing research examining long-term condition self-management support in the context of Twitter. There is also a need for future research to conceptualize how best to support those wishing to utilize these resources in their self-management strategy (eg, computer literacy, resource navigation, and training). Additionally, interventions that seek to better engage the lay natural helpers and super users present in these communities could allow us to understand and use this underutilized resource.

The process of group formation in these online worlds appears to be wholly underexplored in the current research literature. It is clear that social characteristics, such as trust and reciprocity, do exist in these online spaces, but far less is known about the process that facilitates them. Additionally, we know little about how the community is created, how issues of brokerage bring new faces into these communities, how people navigate the mass of communities online to pick one that is suitable to them, or what specific features of an online community they see as important (ie, the presence of a moderator, charity run, professional recognition, site architecture). A case study specifically looking at the social processes within these groups could illuminate this and a longitudinal approach would allow us to see how the relationships in these communities evolve over time.

Because many of the papers involved in this review used methods that did not directly engage those using these communities, there is potentially a bias toward the sharing of positive experiences. There is a need for future research to directly engage with members of these communities to find out why people are reluctant to post and illuminate how these communities help people manage their condition in daily life. Such research would also allow us to further develop our understanding of illness work online, while also helping us better understand such work in the context of preexisting offline support.

## Abbreviations

AMED: Allied and Complementary Medicine Database
CASP: Critical Appraisal Skills Programme
CINAHL: Cumulative Index to Nursing and Allied Health Literature
HCP: health care professional
NHS: National Health Service
